# Implementation of an Intelligent Exam Supervision System Using Deep Learning Algorithms

**DOI:** 10.3390/s22176389

**Published:** 2022-08-25

**Authors:** Fatima Mahmood, Jehangir Arshad, Mohamed Tahar Ben Othman, Muhammad Faisal Hayat, Naeem Bhatti, Mujtaba Hussain Jaffery, Ateeq Ur Rehman, Habib Hamam

**Affiliations:** 1 Computer Engineering Department, University of Engineering and Technology Lahore, Lahore 54000, Pakistan; 2 Department of Electrical & Computer Engineering, COMSATS University Islamabad Lahore Campus, Lahore 54000, Pakistan; 3 Department of Computer Science, College of Computer, Qassim University, Buraydah 51452, Saudi Arabia; 4 Department of Electronics, Quaid-i-Azam University, Islamabad 45320, Pakistan; 5 Electrical Engineering Department, Government College University, Lahore 54000, Pakistan; 6 Faculty of Engineering, Uni de Moncton, Moncton, NB E1A 3E9, Canada; 7 International Institute of Technology and Management, Commune d’Akanda, Libreville P.O. Box 1989, Gabon; 8 Spectrum of Knowledge Production & Skills Development, Sfax 3027, Tunisia; 9 School of Electrical Engineering, Department of Electrical and Electronic Engineering Science, University of Johannesburg, Johannesburg 2006, South Africa

**Keywords:** Regional Convolution Neural Network (RCNN), Multi-Task Cascaded Convolutional Neural Networks (MTCNN), Regional Proposal Network (RPN), Convolution Neural Network (CNN), Discriminative Deep Belief Network (DDBN)

## Abstract

Examination cheating activities like whispering, head movements, hand movements, or hand contact are extensively involved, and the rectitude and worthiness of fair and unbiased examination are prohibited by such cheating activities. The aim of this research is to develop a model to supervise or control unethical activities in real-time examinations. Exam supervision is fallible due to limited human abilities and capacity to handle students in examination centers, and these errors can be reduced with the help of the Automatic Invigilation System. This work presents an automated system for exams invigilation using deep learning approaches i.e., Faster Regional Convolution Neural Network (RCNN). Faster RCNN is an object detection algorithm that is implemented to detect the suspicious activities of students during examinations based on their head movements, and for student identification, MTCNN (Multi-task Cascaded Convolutional Neural Networks) is used for face detection and recognition. The training accuracy of the proposed model is 99.5% and the testing accuracy is 98.5%. The model is fully efficient in detecting and monitoring more than 100 students in one frame during examinations. Different real-time scenarios are considered to evaluate the performance of the Automatic Invigilation System. The proposed invigilation model can be implemented in colleges, universities, and schools to detect and monitor student suspicious activities. Hopefully, through the implementation of the proposed invigilation system, we can prevent and solve the problem of cheating because it is unethical.

## 1. Introduction

One of the best schemes of evaluating and figuring out student capability, wisdom, intelligence, expertise, and knowledge is an examination in academic institutions. There are various methods to estimate the student capabilities of students, such as projects, written examinations, presentations, assignments, and oral examinations. In a traditional and formal examination, question papers are provided to students where they respond in the form of answers in a limited time period. The invigilators’ (examination supervisors) duty is to prevent any kind of communication, such as gesture communications, whispering, and movements during the examination and restrain students from cheating and prohibit the use of notes or any cheating materials. To monitor students during examinations, each room requires a head invigilator, who will ensure that the exams are conducted with honesty and who will sort out any problems that may occur during the exam. A supervisory committee is also organized to check and invigilate all the exams rooms at different time instances. A separate invigilator is required for approximately every 50 students in the examination center. To overcome the problem of offline examinations monitoring and to reduce a load of invigilation on supervisory committee members, we proposed a model based on deep learning algorithms of computer vision that can detect and recognize people performing any suspicious activity i.e., neck movements during the examinations. 

Throughout the world, these exams get monitored by human invigilators. Today all high-stakes examinations have a high incidence of cheating. The main objectives of this research are:

▪ To reduce academic dishonesty and cheating among the students during examinations.
▪ To monitor and capture the prevalence of academic dishonesty among the students in the higher education context more precisely and accurately.
▪ To reduce the burden on the Invigilation staff members.
▪ To identify the students interpreting any suspicious task through the face recognition module.
▪ To generate a report in which all students’ names are written along with the percentage of cheating activity.

Presently, there is no invigilation system that can detect and analyze the suspicious activity of students during examinations based on RCNN along with face recognition algorithms. The proposed model for invigilation implemented two deep learning modules: one is Faster RCNN; and the other is MTCNN. The dataset used to trained the model is completely self-generated in which students exhibit cheating and non-cheating activities. The proposed system is totally software-based without using any hardware except the camera for live video recording of students, and exhibits an accuracy of 98.5 percent in detecting the unethical activities of students with the monitoring capacity of 100 students at a time. Many automated invigilator assignment systems have been introduced worldwide. These systems can monitor the student’s actions during online examinations using a web camera [1,2], which requires an independent automated system for every individual. The existing Invigilation systems are based on image processing techniques [3] and computer vision [4], and are used to supervise the offline written exams, but they cannot monitor more than 15 students at a time. To the contrary, the proposed invigilation system is capable of detecting and capturing more than 100 students. An automatic invigilation system has been designed throughout the world to monitor and supervise students efficiently during examinations. The automation of traditional exam invigilation using CCTV and bio-metrics [5] has been implemented in Bangladesh to monitor students during examinations. To monitor students’ cheating activities CCTV is being used. To check verbal communication microphones, are implemented. All the hardware used in this system ensures that there will be the minimum possibility of any suspicious activities in the exam. A biometric system that is implemented in this system will make sure that only authorized and registered students sit in the examination hall. An intelligent invigilator system based on artificial vision [6] is also designed and implemented. 

In this research, a system is designed to enhance the quality of the video of the invigilation area by minimizing the packet loss to improve the overall capturing capacity of the system. The system consists of both hardware and software parts in which analogue-to-digital conversion circuits, correction circuits, and different multiple circuits are used. An automatic invigilation system based on an adaptive threshold [7] is designed to capture and draw out features of suspicious activities, and a system is implemented which recognizes human body contours. 

All automatic invigilation systems that are described above perform their tasks efficiently and give appropriate results, but the problem with these systems is that they are based on both software and hardware parts which makes them costly and unreliable. The hardware also requires high maintenance to operate effectively. The proposed model is based on the software part and represents appropriate results with higher accuracy. On the other hand, the proposed system is fast compared to other systems as it requires less computation time. In the proposed model two deep learning modules are implemented in which Faster RCNN is used for cheating activity detection and MTCNN is used as face detection and recognition. In this model, a report is generated for 1 h, which is the limited time allotted to students to attempt the paper in which against each student name a percentage of cheating and no cheating activity is mentioned. A timer of 3 s is used to detect the student behaviour, and if the student is continuously moving his neck in order to peek at another student’s paper then it is labelled as a cheating activity. 

The drawback of the proposed system is that faster RCNN rectangular boxes are used to detect students, so there is a probability of overlapping of detection boxes when students’ sitting arrangement is conducted. 

**Figure 1:**
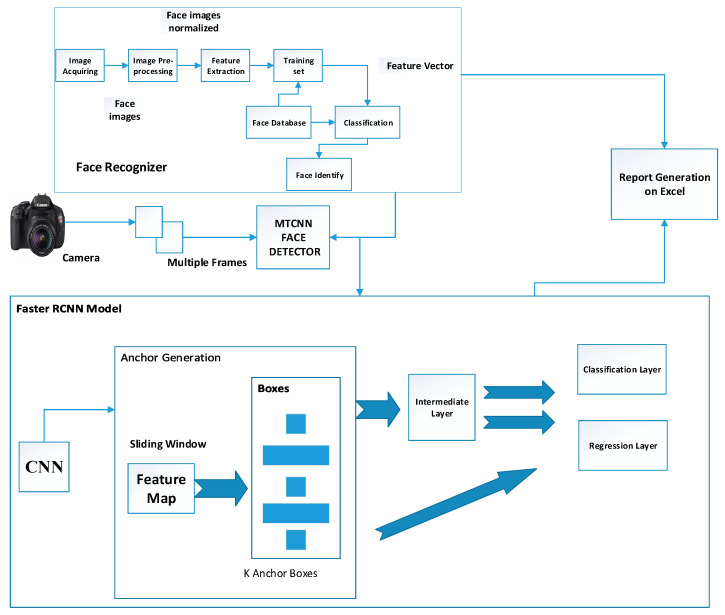
The Proposed Model Overview Diagram.

To resolve this issue mask RCNN can be used instead of faster RCNN in which students are being detected through masking in which there is less chances of detection overlapping and that makes the system more accurate. The proposed model overview diagram is shown in Figure 1. 

This paper has been structured as follows: in Section 1 introduction, Section 2 consists of a literature review related to traditional invigilation systems. Section 3 consists of the methodology in which the proposed model is being explained and in Section 4, the model implementation is elaborated on, an the evaluation measures and results are discussed. Section 5 and Section 6 consist of the conclusion and future recommendations. 

## 2. Literature Review

Numerous systems are proposed in the existing literature on inactivity detection of humans and video surveillance. For the detection of human activities, the general network includes various steps like motion detection, background and foreground modeling, segmentation, classification, object tracking, and the identification of the person’s behavior & activities along-with person face recognition. Students and examination supervisors, by using their smartphones or computers, can access a web application called the Examination Management Automation System. These systems have various details in modules such as students details, staff details, and hall details with proper explanations and descriptions [8]. 

Xiao, H. [9] proposed an automatic real-time-based invigilation system by using a single-shot Multibox (SSD) detector and comparing the model accuracy with yolo and other object detection deep learning modules. The accuracy achieved by implementing this model to detect student illegal activities during examination is 79.8%. In their research work, Malhotra, M. [10] implement YOLOV3 to detect and identify illegal activities of students during the exam with an accuracy of 88%. It is worth noting that YOLOV3 turned out to be efficient in several other applications [11,12]. It has been adapted to the education sector. 

In their research, Adil, Md. [13] proposed a model that will help schools and universities monitor and detect various suspicious or unethical activities like whispering or hand contact, etc. in the classroom during examinations. The system detects hand-contacts of students, identifies and detects students who are looking at another’s answer sheet. The methodology is based on certain threshold levels, a certain threshold or grid is formed around the student and whenever a student moves his hand beyond this level, it will be detected. They used the voila jones algorithm and the Adaboost algorithm for their proposed model. Based on color and grid formation, the direction of the head and hand contact is detected, and the face is recognized by using the voila jones algorithm. The drawback of this algorithm is that it requires multiple cameras to capture students from different angles which requires high processing power and grid overlapping may occur when there is less space in students sitting arrangement. 

Kulkarni, Rutuja [14] proposed a method in which Inception V3 CNN [15] is applied for the classification of body movements into legal and illegal activities during the examination. In this paper, the automatic invigilation system was proposed in which students are being monitored and the backbone of this architecture is the inception V3 CNN model. But the problem with this invigilation system is that it is enables the capture of more than 15 students during the examination while live streaming, so it failed to be implemented in large examination centers where student numbers exceeded 100. Pandey, I.R. [16] in their research paper, proposed a face recognition system in which CNN is implemented for feature extraction and person classification Softmax is used. Jiang, H. [17] proposed a face detection and recognition model by using faster R-CNN. They train a Faster R-CNN Face detection model by using WIDER face dataset and in another research paper, they have proposed a face detection model which is based on an evolutionary Haar filter [18]. In the training dataset set, there are 12,880 images and 159,424 faces. They describe randomly sampled images of the WIDER dataset. Viola jones algorithm [19] is also being used for face detection and identification. The Convolution Neural Network (CNN) was used for feature xxtraction and then these features were passed to DDBN for activity detection. Zhenhong, Wan [20] represents a deep learning module for the identification of cheating behaviour during examinations. An object detection algorithm YOLO is implemented to detect the boundary box for each student along with that another algorithm which is open pose used to identify and recognize student correct posture and position and label them as suspected or non-suspected. A deep learning algorithm that used as a binary classifier in this model get trained on the training dataset. The training dataset consists of only two classes of cheating: peeping in to another student paper, and sharing or exchanging answer sheets. For testing the speed and accuracy of the proposed model, a surveillance camera is used to make a video of students during examination, the inter-frame difference method is also implemented to enhance the detection speed and accuracy of the proposed model to extract multiple frames or images from the surveillance video and then pass these images to the model for student cheating behaviour detection. Md, Abdul [21] developed an invigilation system which is based on the Internet of things(IOT) to supervise the students in Examination Hall with a camera in order to reduce the cheating activities in an examination hall. The IOT hub is connected to camera, the database system of the university and also to the examination department to monitor the students’ activities during the examinations. It is important to monitor students online via camera because sometimes teachers cannot catch students who are cheating through different tricks and techniques. To improve the monitoring system of students during invigilation they proposed this model that is IOT based. D, Binu [22] proposed an Automatic Authentication Examination System in which students verification is done by Automatic face recognition through deep learning module and fingerprint Identification. Face detection can be done through deep learning based object detection models is the process used for the recognition of students through images. In the proposed model, the database in which images of all the students from different angles and fingerprint samples are stored is created and then every student face and fingerprints get mapped with the database data to check whether a student is eligible candidate or not. If the student face and fingerprint features match with the existing database, then the student is eligible to attempt the paper. 

S, Ketab [23] proposes an Invigilation system with the authentication process of students. The proposed model is a smart authentication and automatic invigilation system for both offline and online examination. Multi-modal Bio-metrics technique is used for the students’ identification along with a 3D facial recognition method. To monitor students during examination, an eye tracker system is implemented to detect suspicious movements of eyes and speech recognition module is implemented to detect any improper voice. Speech recognition is a deep learning module which is trained on speech pattern to detect specific voice note. Kavya, Sri [24] proposed an automatic invigilation system which is web-based, a secure website is designed for an online invigilation system in which for the front end CSS and HTML languages are implemented to design a secure website and an msql database is used for the back-end design. PHP is also used as a programming language in this project. A detailed comparison of existing research and the proposed work are presented in Table 1. 

Yunjie, Fang proposed an Automatic Invigilation system. Ratul, Prosad [25] proposed an Automatic Invigilation Management and class schedule system in which invigilators and teachers’ duties are divided equally for invigilation and the system is based on a greedy approach. The proposed method has two main parts to Automate class routine and schedule system one is Admin panel and the other one is user panel in which all teachers’ information, room allocation and schedule can be seen and altered according to the requirement. Exam Automatic Invigilation system can be implemented in both python and in java-script. Yang et al. [26] in their proposed model elaborate the method of Faster R-CNN in detecting different running patterns like running, walking, escaping, etc. These patterns are presented for a single or multiple individuals. An Automatic System is being proposed by Rahmad, N.A. [27] to locate the position of badminton players in the game by using Faster RCNN. For the dataset, multiple videos of badminton players are converted into frames (Images) that are used for training and testing purposes. Suspicious Activity has been detected by using the Discriminative Deep Belief Network (DDBN) proposed by Scaria, E [28]. Yousafzai and coauthors examine the deep neural network model, namely, for the purpose of efficiently predicting student performance from historical data [29]. They used the attention based Bidirectional Long Short-Term Memory network. 

## 3. Methodology

In the proposed methodology an Automatic Invigilation System is designed and implemented to capture Student unethical activities during an offline examination. Deep learning model Faster RCNN is implemented as a binary classifier to classify students in cheating and no cheating categories based on their head orientation. The MTCNN model is used for student face recognition and then the results of both modules are combined to generate student status report. As mentioned before, the proposed model is used for monitoring student activities during Examination. The unethical is classified as cheating. The classification is based on head orientations. A cheating label is considered for the following head movements Left, Right, Upward or while peeking into other paper, Backward and No cheating label is considered only for down movement of the head when the student is doing his/her Exam. 

### 3.1. Image Acquisition

In data Acquisition camera is used to capture the video of students, and then the video is converted into multiple frames [images] to detect and recognize the students. Table 2 shows the training dataset Types and description of different images taken during experimentation. 

A separate dataset has been collected for suspicious activity detection and face recognition. For monitoring head orientation, Training and Testing datasets are created for students moving their heads left, right, up, down, backward and generate a “cheating”and “No cheating” label manually on these images by using label image software. 

LabelImg software is implemented for the annotation of training dataset and generation of .xml file, as after annotation of image the boundary box 4 points stored as .xml file. Table 3 gives an illustration of how to annotate images through VGG and IabelImg software both are used for image Annotation. The dataset contains 5000 images in which 1000 images are single, and 4000 images are of different classrooms in which students are performing cheating and No cheating activities. 80% percent of the dataset is used for training and 20% for testing purposes. CSV file is generated through xml_to_csv. py python code in which each object label is defined with its bounding box values. After the generation of test_labels. csv and train_labels. csv files, T_f_ records are being generated through which the model has been trained. We trained our faster RCNN model on a labeled head oriented dataset. After training, the model is tested on live video of an examination hall from an automated surveillance camera. In implementation first, the video is converted into frames and each frame is checked for head movement and the head movement is classified as cheating or not cheating movement. A separate database of known faces has been created for students’ face identification. The dataset contains 1000 images of students where every student’s front, left and right angles of the face have been captured. 

### 3.2. Face Detector

The face detection MTCNN [30] is implemented to detect students’ faces. The algorithm detects human faces along with 5 face landmarks through bounding boxes and it consists of 3 stages, firstly multiple windows are generated across a person’s face and then more complex CNN are applied to discard all the windows having no faces and at the third stage the more advanced CNN is implemented to detect face landmarks and to refine windows. In the proposed invigilation system, the algorithm MTCNN successfully detects all student faces. 

### 3.3. Proposed Model

**Figure 2:**
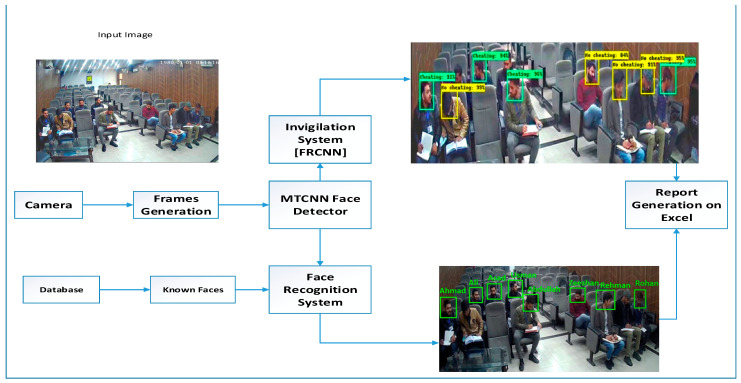
Proposed Methodology of the Invigilation System.

In the proposed model two modules are implemented: the first is the Object detection API module in which Faster RCNN is used as a classifier and the second is the Face recognition module. In the Faster RCNN inception module, the model gets trained on the invigilation dataset and then tested. It is implemented as a binary classifier. Face Recognition Module is used for student identification. Statistical Report is generated through the integration of the face recognizer and a classifier. The proposed Methodology of the system is shown in Figure 2. 

In the proposed model surveillance camera is used for live video recording of students in the Examination Hall. Video is then converted in to frames by using python code in which after every 0.05 s frame is extracted from the video. The Extracted frames are then inserted into the Multi-Task Cascaded Convolutional Neural Network (MTCNN) for face detection. MTCNN is a deep learning model used for face and facial features detection with more than 95% accuracy. It has 3 stages of CNN to detect full face along with 5 facial landmarks. After face detection the Image is inserted in two deep learning models in which one is Faster-RCNN and the other is Face Recognition Model. Faster –RCNN is an object detection module, in this project we implement it to detect unethical or cheating activities in the examination hall. The model is trained on two types of images one is labeled as Cheating and the other one is labeled as no Cheating. Face Recognition module is used for the identification of students in which each student face embedding are get mapped with all the face embedding available in the dataset. At the end, the overall report of all the students is being generated in which against each student name percentage of cheating and no cheating activity is mention.

#### 3.3.1. Faster RCNN Model

Faster R-CNN [31,32,33] has two parts. The first part is a fully connected network known as the Regional Proposal Network [34] (RPN), which generates regional proposals which are further used as an input for the second part of the model. Fast R-CNN detector is a second part that classifies each Region of Interest. Convolution Neural Network is implemented for feature extraction of an image. Figure 2 shows the proposed methodology flow diagram of the implemented invigilation system. 

These image’s features are taken as an input for a Region Proposal Network (RPN) and generates a set of rectangular proposals with the corresponding Objectiveness score. To generate region proposals, a small sliding window is mapped on the convolution feature map which is the output of the last shared convolution layer. 

**Figure 3:**
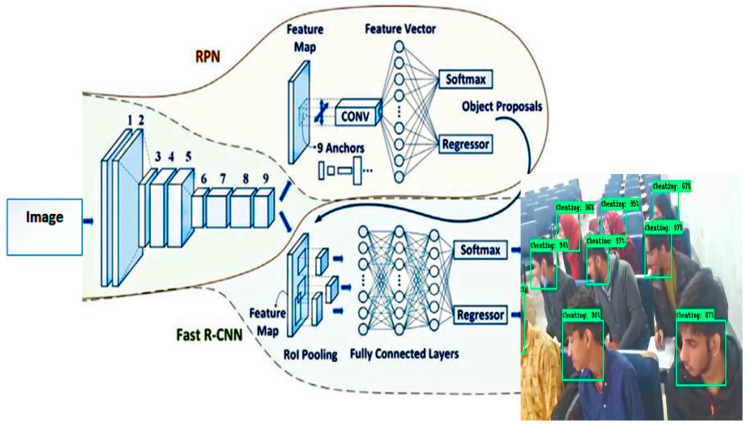
Faster RCNN and RPN.

A set of regional proposals are fed as an input to a Faster RCNN detector. Each proposal is passed through a ROI (Region of Interest) pooling layer which generates a feature map of fixed dimension. The fully connected layers (FCs) mapped these features into feature vectors. These feature vectors are the inputs of the box-regression layer (reg) and a box classification layer (cls) for the classification process. The Softmax classifier is used in a faster RCNN model. In Figure 3, the framework of Faster RCNN is represented in which there are total 9 Anchor boxes used to extract features from the input image.

#### 3.3.2. Features Extraction

The features are extracted from VGG16 architecture. ImageNet dataset is used to train VGG16 in order to extract features from the required image. There are in total 15 convolution layers in the VGG16 architecture to extract features, three fully connected layers to train the network on the extracted features and one Softmax classifier. There are 64 filters in first two convolution layers, 128 filters in the 3rd and 4th layers and 256 filters are present in the 5th and 6th convolution layers. After every convolution layer max pooling having filter of (2 × 2) is applied to reduce the dimensionality of features. 

#### 3.3.3. Region Proposal Network (RPN)

RPN accepts the features maps produced by the VGG16 model and proposed multiple regions of the features map. RPN uses Feature maps generated through the backbone of Faster RCNN and then generates regions of Interest (ROI). There is also a binary classifier in RPN which classifies each anchor as a background region (an anchor containing the background part of an image) and a foreground region (an anchor containing the foreground part of an image). Anchors that are classified as foreground regions and contain most of the objects are considered as a region of interest and then after ROI Pooling are passed to the object classifier through fully connected layers (FC). In the RPN, anchors are generated to extract proposals from the features of the input image and anchors are classified as foreground or background anchors by comparing them with the ground truth box to eliminate background ones and it also generates object/no-object file probability that is further associated with each anchor. In RPN Non-Maximum Suppression technique is implemented to reduce the overlapping between anchors and redundancy to choose the best regions having high objectness probability. Objectness probability is probability of objects existence in that particular anchor box. 

#### 3.3.4. IOU and Non-Maximum Suppression

The technique IOU (Intersection Over Union) computes the intersection level between the predicted bounding box and the ground truth bounding box. While the IOU value is equal to 1, the prediction box is perfect and has a maximum number of objects in it. In background or foreground classification, anchors whose IOU < threshold value are considered as negative anchors and are discarded. The formula to calculate the IOU is shown in (1).

(1) $$\mathrm{IOU}=\frac{Area\;of\;the\;intersection}{Area\;of\;the\;union}$$

#### 3.3.5. RPN Classifier

The role of the classifier layer in RPN is to detect the good or bad proposals for image object class recognition. It does not recognize the class of objects within a region. The classifier also defines the score of the probability of objects within a region to choose which one is the best. The main task is to classify anchors as background whose IOU < 0.3 among the predicted bounding box and the ground truth table and to classify the anchors as foreground regions having IOU > 0.7. 

#### 3.3.6. ROI Pooling

RPN generates regions of different size. ROI pooling layer is implemented to reduce the dimensionality of the regions in order to make them same in size. ROI pooling layer is connected to fully connected layer and provide uniform regions of proposals generated by RPN. 

#### 3.3.7. Classifier and Bounding Box Regressor

The main purpose of the bounding box Regressor is to further refine the coordinates for the bounding box once the object has been classified through an SVM or Softmax classifier. In case the object cannot properly fit within a bounding box generated by the classifier then the bounding box Regressor is implemented to adjust the 4 offset values of bounding box to adjust that object properly within generated bounding box. 

**Figure 4:**
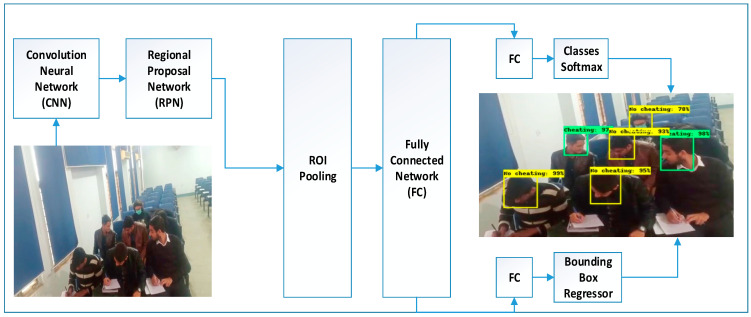
Faster RCNN for Suspicious Activity Detection.

For the classification purpose, mostly support vector machines (SVM) and Softmax are implemented to classify and detect multiple objects within an image via bounding box. TensorFlow’s Object Detection API Repository is used to train the object detection Classifier. The model Faster-RCNN-Inception-V2 model is trained on the invigilation dataset with a training accuracy of 99.5% and testing accuracy of 98.5%. Around 80% of the whole dataset is in the training folder and 20% is in a testing folder. Figure 4 demonstrates that how Faster RCNN implemented for unethical activity detection. 

### 3.4. Face Recognition System

For the identification of students, face Recognition with Open-CV [35] is implemented. Firstly, the students’ faces are detected to recognize them. MTCNN [36] is used for face detection. Face embedding models are used to extract the facial features of students. A vector named face embedding represents the facial features of the student and is used for recognition and identification of the student. A separate database is created for student identification. In the system, there is face embedding for each student. In a live video streaming each student, face embedding has been calculated and then compare with the known face embedding that is already in the dataset. 

## 4. Model Implementation

### 4.1. Experimental Setup

Faster rcnn inception file is used to train the model. During training, the total loss starts at 2.413 and then gradually decreases as the number of training steps increases and ends at the loss of 0.390–0.134 at the 50–60 k steps. The learning rate is in the range of 0.002 to 0.0002 and the batch size is 1. 

The algorithm used for learning is stochastic gradient descent. 6 h was required to train the faster RCNN classifier on the training dataset on Linux Ubuntu 16.04 by using graphic processing unit (GPU) with the training accuracy of 99.8% and we assure that the classifier accuracy will be more than 95% on the testing dataset. 

### 4.2. Evaluation Measures

The Faster RCNN is jointly trained with 4 losses Region Proposal Network (RPN) classification (Object foreground/background) loss, RPN Regression loss (Anchor → ROI). The fast RCNN Classification loss (object classes) and Fast RCNN Regression loss (ROI → Bounding Box). The goal of the RPN is to make things more understandable to the overall network. Features from Convolution Neural Network (CNN) are fed to the RPN network and anchor boxes are being generated. The role of the classifier in RPN is to classify boxes into two categories (Foreground or background anchor boxes). The bounding box Regressor in RPN is used to refine the offset values of foreground anchors to make sure that the objects are fully visible. 

Both classification and Regression loss contribute to the RPN loss. The function of Cross-Entropy is used to estimate the classification loss of RPN and a distance function in which the distance between the regression coefficients of the ground truth foreground box and the coefficients foreground anchor box is generated by RPN while (2)–(4) shows RPN path loss, classification loss and Bounding Box Regression loss modeling, respectively.

RPN Loss = Classification Loss + Bounding Box Regression Loss(2)

Classification Loss:

cross entropy (predicted_class, actual class)(3)

Bounding Box Regression Loss:

(4) $$L_{\mathrm{loc}}=\sum_{u\in \mathrm{all}\;\mathrm{forground}\;\mathrm{anchors}}l_{u}$$

**Figure 5:**
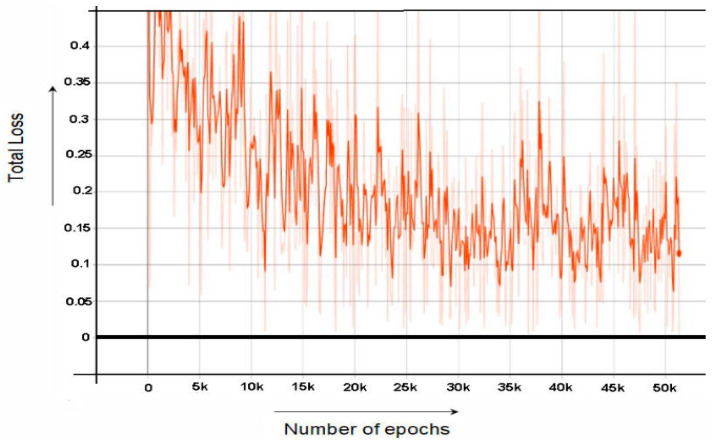
Faster RCNN Classification Loss.

Similar to the RPN Loss, classification layer loss has two components classification loss and bounding box regression loss. The classification model (faster RCNN) loss consists of two losses, one is classification loss and the other one is regression loss. The classification loss is basically a difference between actual value and the predicted value. Bounding box Regression Loss is the sum of all loses related to foreground anchors. Foreground anchors are those bounding box who contain foreground images. The graph of Faster RCNN Classifier loss is shown in Figure 5. In all the graphs, we have considered the trend of dark orange lines and neglect the other lines.

Classification Layer Loss = Classification Loss + Bounding Box Regressor Loss(5)

The RPN classifier is a binary classifier that classifies images as foreground or background image and the model classifies different objects within the image and the number of objects that the model classifies depends upon the number of classes on which the model gets trained. The classification loss is calculated as Entropy loss in which actual class and predicted class of objects are used as parameters. The formula to estimate cross entropy loss is shown in (6) in which ‘N’ is the total number of samples divided by the sum of all the log function where i is the total number of Anchor boxes.

(6) $$\mathrm{Cross}\;\mathrm{entropy}\;\mathrm{loss}=\frac{-1}{N}\left(\sum_{iℰAnchor\;Box\;samples}\log\frac{e^{x\left[i\right]\left[\;c_{j}\right]}}{\sum_{j}e^{x\left[i\right]\left[j\right]}}\right)$$

**Figure 6:**
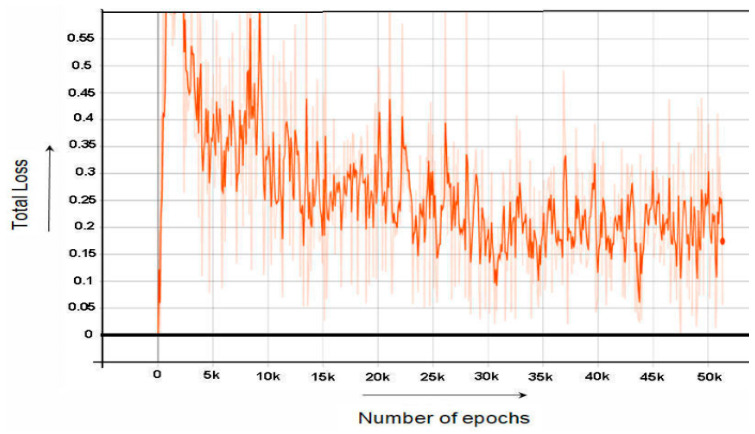
Faster RCNN Localization Loss.

The bounding box regression loss in the classification layer estimates the regression coefficients of each correct class and compares it with regression coefficients of the ground truth bounding box. Figure 6 shows the graph of Faster RCNN bounding box Regressor loss is shown.

**Figure 7:**
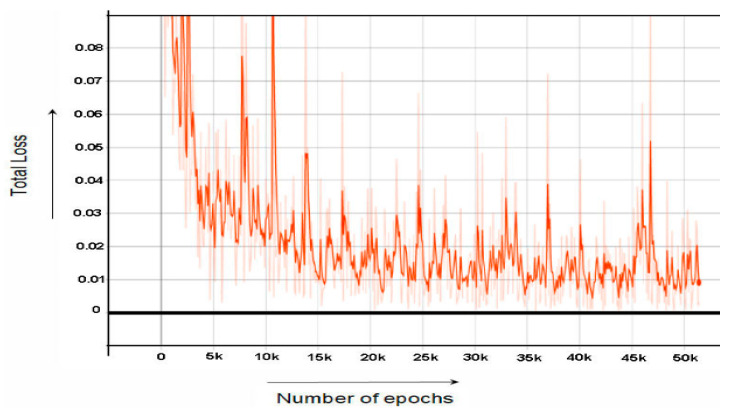
The representation of the RPN Objectness Loss.

**Figure 8:**
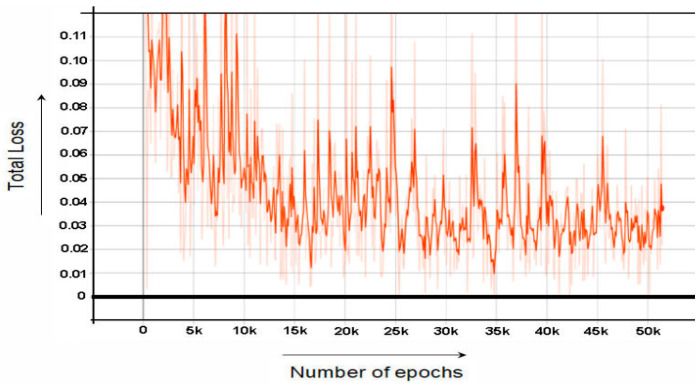
RPN Localization Loss.

**Figure 9:**
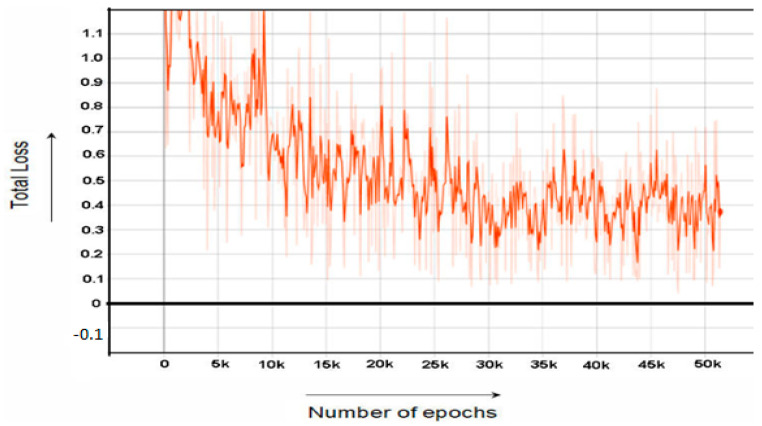
Total Loss vs Number of Epochs.

While Figure 7 and Figure 8 shows the graph of RPN Classifier loss and graph of RPN Bounding Box Regressor loss, respectively. Figure 9 the graph of total loss is represented. The faster RCNN classification loss starts at 0.61 and gradually decreases to 0.018 after 60,000 epochs and after 60,000 epochs the loss graph becomes straight. Similarly, the RPN classification loss starts at 0.55 and after 60,000 epochs end at the loss of 0.01. 

The Testing accuracy can be defined as the percentage of correctly classified instances. Testing accuracy is the percentage of correctly identified instances. The formula to estimate accuracy is shown in (7).

Accuracy = (TP + TN)/(TP + TN + FP + FN)(7)

The faster RCNN model testing accuracy on this invigilation dataset is 98.5 as the model correctly identified 988 images from 1000 images. 12 images are misclassified as 8 images are categorized into wrong class or label, 2 images identified inappropriate regions of interest (ROI) and the other 2 images have both issues misclassified and have the wrong ROI. The confusion matrix is also described in which TP, TN, FN, and FP are defined. The performance of the proposed model depends on its accuracy and the capacity of model to capture students in one frame at a time. The model Accuracy on detecting cheating and no cheating is 98.5% when there are 100 or less than 100 students in a classroom. The model accuracy decreases as the number of students exceeds the limit of 100, as the model Accuracy is 94% when the number of students in a classroom is 150 beyond the limit of 100 because now the other 50 students may be classified incorrectly, or some may not be detected by the model. 

The model is the best fit when there are 100 students in an examination hall. The dataset is totally self-generated, and the training images contain almost 10–15 students in each single image and student group images are taken from both front and back. The model also capable enough to monitor those students who are seated in the back of room and classify them in a correct category. In Table 4, hyper parameters of the faster RCNN model is being discussed. 

Table 5 displays the confusion matrix on 1000 test images is represented. Table 6 shows the accuracy of Faster RCNN model is described.

## 5. Results and Discussion

### 5.1. Faster RCNN Results

**Figure 10:**
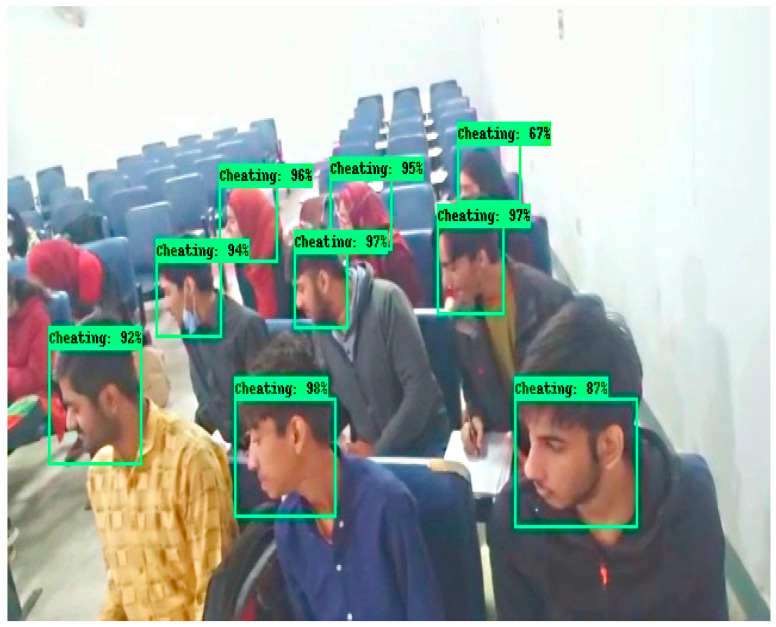
Results of invigilation system in classroom.

**Figure 11:**
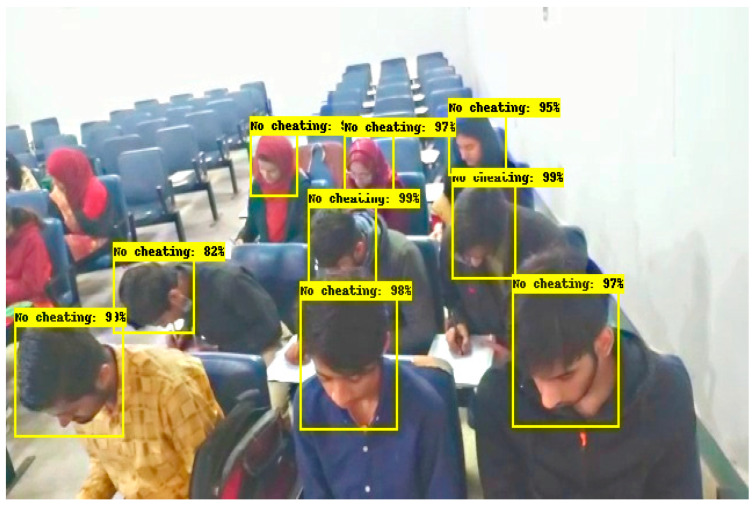
Results of invigilation system in the classroom.

The results shown in Figure 10 and Figure 11 are obtained in a classroom where 9 students are doing their papers. In Figure 10, all students’ neck orientation is in the left direction and the classifier detects and classifies all students in a cheating category. In Figure 11, all students are doing their paper and their neck orientation is in the downward direction, so the classifier detects and classifies each student with a No cheating label. The classifier Faster RCNN correctly detects and identifies these types of images with a minimum error rate. 

**Figure 12:**
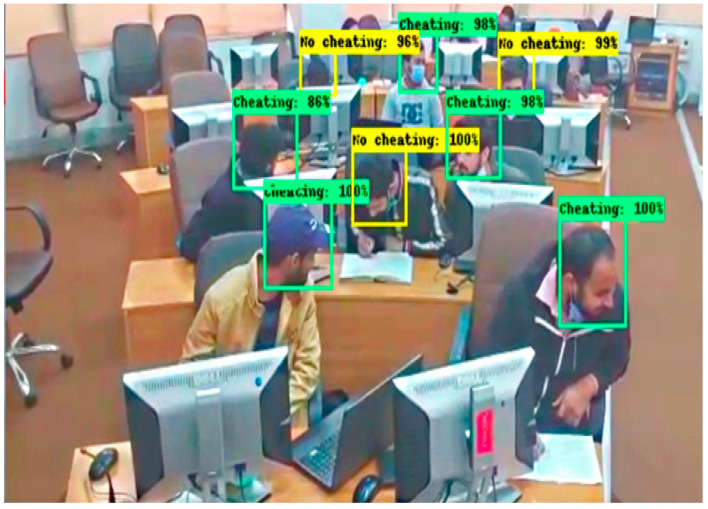
Result of Faster RCNN in Computer lab.

**Figure 13:**
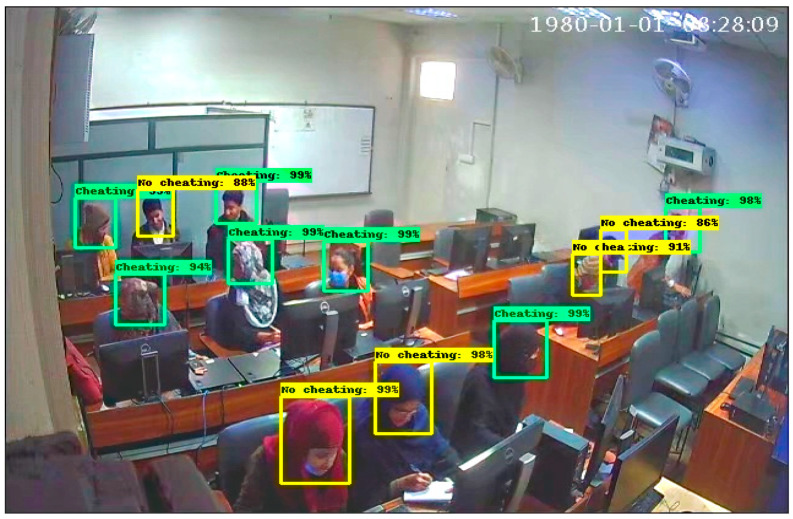
Result of Faster RCNN in lab.

**Figure 14:**
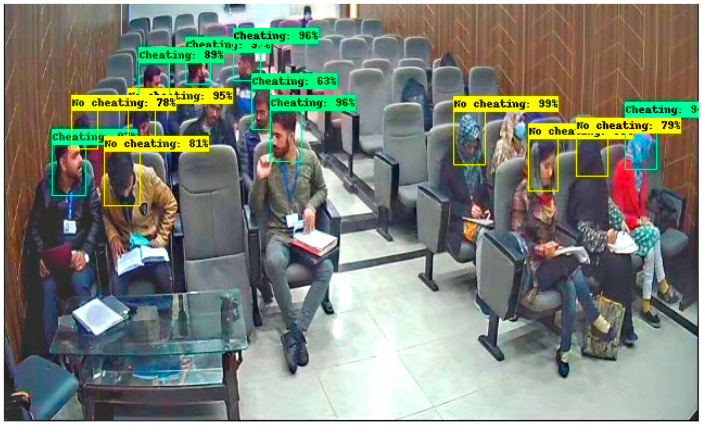
Result of Faster RCNN in Seminar Hall.

**Figure 15:**
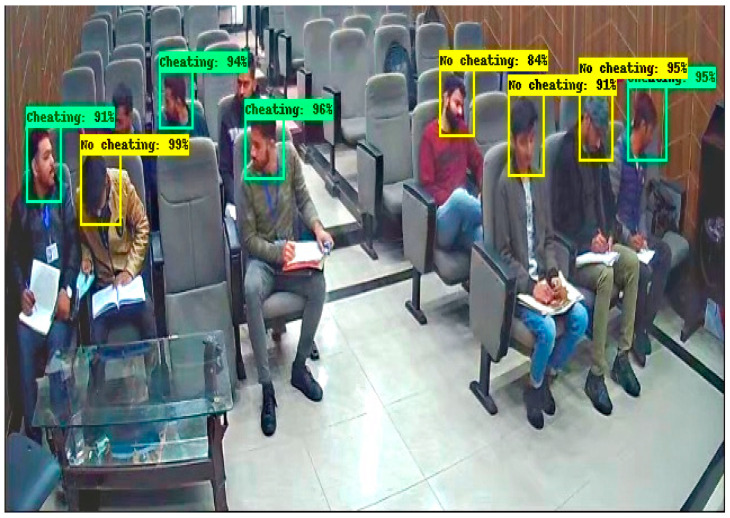
Result of Faster RCNN in Seminar Hall.

These results are captured from the computer lab in which some students are doing their paper, and some are trying to peek into another paper by moving their necks. In Figure 12, there are 9 students in which 3 are categorized into No cheating label and 6 are categorized into a cheating label. In Figure 13, there are 12 students in which 5 students are classified with a No cheating label and 7 are classified with a cheating label. These results are obtained in a seminar hall where students are doing their papers during the exams for a limited period of 30 min. In Figure 14, there are 15 students in which 6 students classified with a No cheating label and others are categorized with a cheating label and in Figure 15 there are 10 students in which, 4 students are detected with a yellow bounding box with a No cheating label and 4 students are detected with a green bounding box with a cheating label. 

### 5.2. Face Recognizer Result

**Figure 16:**
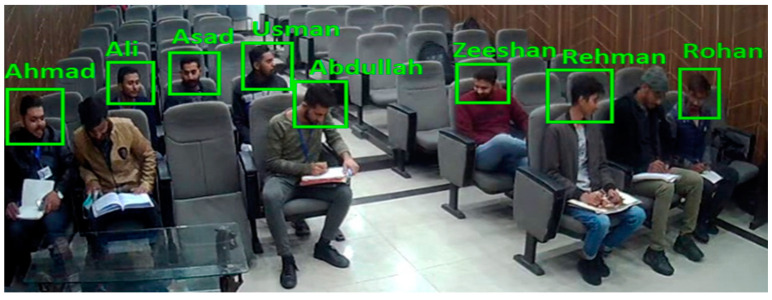
Result of Face Recognition Model.

The MTCNN model is implemented for students’ face detection and Recognition. In face recognition, the database is created in which all student’s face images and information is stored and for each student, face embeddings are being matched with all the face embeddings stored in the database for face identification and recognition. In Figure 16, results of Face Recognition Module are represented. 

### 5.3. GUI Interfaces

**Figure 17:**
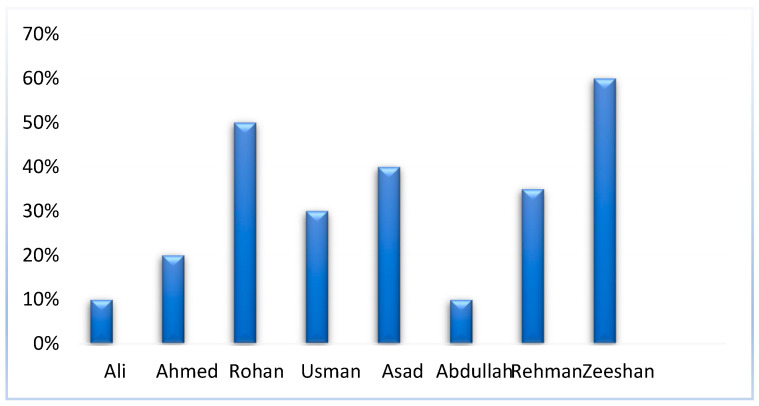
Report graph of Students.

The student names are recognized from the face recognition module, and students’ activities are detected during examinations like cheating or No cheating from Invigilation System. Figure 17 shows the report of students generated through the results of two deep learning modules is represented. In the face recognition module, face detection is done through MTCNN, and after detection, features are being extracted. The extracted features are then compared with the known features to identify the student names. In the invigilation system deep learning model faster RCNN is implemented which is trained on the invigilation dataset to identify any suspicious activity during the exam. The report of 30 min is being generated through the combination of both these modules. Each student is continuously being monitored or checked during the exam and at the end, the overall percentage report of students is being generated through the proposed system. In the report, each student’s cheating percentage along with his/her name is mentioned. 

The limitation of the proposed model is that it considers only the head orientation of the students to identify Cheating or No Cheating activity because the model Faster- RCNN is trained on the student’s head-oriented dataset where student ‘head down’ movement is considered as No cheating and student left, right, up movement of head consider as Cheating activity. The proposed Invigilation system can be further improved by the training of a faster RCNN system to detect hand gestures and hand contact while passing sheets by extending the existing dataset including the classes of hand gesture images and hand contact images of students. It can also be used to detect any kind of destructive objects like calculators and phones the examination to minimize the possibility of cheating in offline examination. Different Deep Learning models like YOLOv4, RCNN, and Mask RCNN can also be implemented to detect cheating activity. 

## 6. Conclusions

Faster RCNN is a deep learning model implemented for object detection and classification. It gives accurate results and better accuracy as compared to Fast RCNN and other CNN models for invigilation purposes. In the proposed model faster RCNN is implemented as a binary classifier to classify student activities into two categories: cheating; and no cheating. The no cheating label is considered for those students who are doing their paper and the cheating label is considered for those students who are continuously looking left, right, and peeking into other student papers for cheating. In this proposed paper, an automatic invigilation system is being implemented to detect unethical activities of students during an examination, deep learning model Faster RCNN is implemented as a classifier that is trained on the Invigilation dataset with the training accuracy of 99.5 and on testing the accuracy of the model is 98.5. Student identification and recognition are done through MTCNN and faces Recognition module with an accuracy of 95%. The results of both the faster runner classifier and face recognition module are combined, and students’ status reports are being generated on excel. The proposed model is better than the existing model as it captures more than 100 students at a time and the computation time to get the desired result is less as compared to other models. The proposed invigilation system can be further improved by the training of a faster RCNN System in detecting hand gestures and hand contact while passing sheets. It is also possible to detect any kind of destructive objects like calculators and phones during the examination to minimize the possibility of cheating in offline examination by using other object detection deep learning modules like YOLOv4, RCNN, and Mask RCNN. 

## Figures and Tables

**Table 1:** Research Matrix Exhibiting comparison of Existing Studies with the Proposed Model.

Title	Method	Hardware	Activity and Movement Detection	Face Recognition	Result
Automation of Traditional Exam Invigilation using CCTV and Bio-metric [5].	Parallel Data Acquisition Tool (PLX-DAQ) for student bio-metric	Microphones, CCTV cameras, Speakers, Fingerprint Sensors	Yes	No	Error < 10%
Realization of Intelligent Invigilation System Based on Adaptive Threshold [7].	Optimized Expectation Maximum (EM) Algorithm with adaptive threshold	Monitoring and seat calibration module with identification Alarm	Yes	No	Error < 10%
Application of SSD core detection algorithm in intelligent visual monitoring of examination room [9].	Single Shot Multi-Box Detector (SSD 300)	CCTV cameras	Yes	No	79.8%
Automatic Invigilation Using Computer Vision [10].	YoloV3 (Only look Once) Algorithm	CCTV cameras	yes	No	88.03%
Automated Invigilation System for Detection of suspicious Activities during Examination [13].	viola jones Algorithm, Ada-boost Algorithm	CCTV cameras	Yes	Yes	Error < 10%
Real-time-Automatic Invigilator using Computer Vision [14].	Inception V3 CNN Algorithm	CCTV Cameras	Yes	No	For head orientation 70% and for face recognition 84%
Proposed Model	Faster RCNN, MTCNN Algorithm	CCTV Cameras	Yes	Yes	98.5% for cheating activity Recognition and 95% for face Recognition

**Table 2:** Training Dataset Types and images description.

Type	Image	Description
A	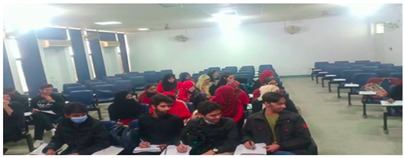	In image type ‘A’, 15 students are looking all of them to the right direction.
B	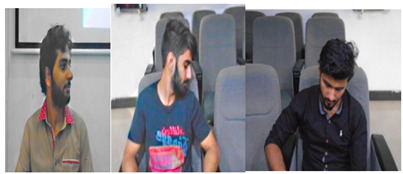	In images type ‘B’ there are individual pictures of the students where they are looking to their left, right, and in a downward direction.
C	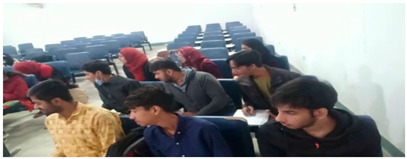	In image type ‘C’, there are 9 students all of them are looking in the left.
D	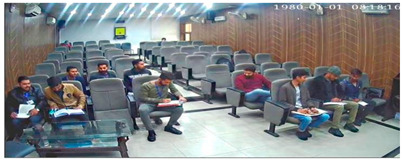	In the image type ‘D’ there are 10 students where some are looking into other students’ papers, and some are doing their paper.

**Table 3:** Training Dataset Annotation.

Type	Image	Description
A	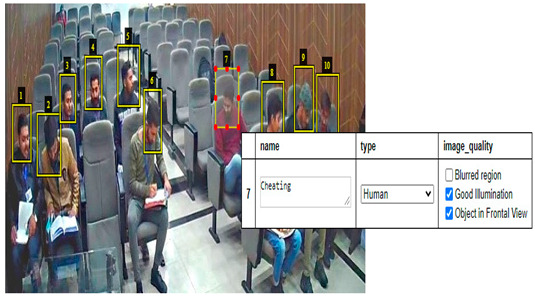	In image type ‘A’, student is looking on his right, labeled as ‘Cheating’.
B	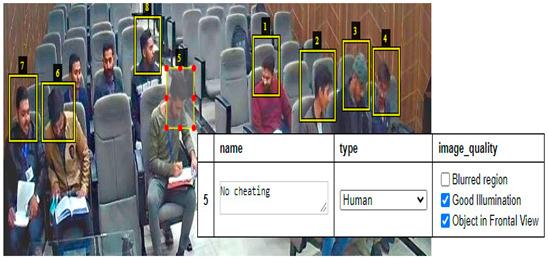	In images type ‘B’, student is busy in doing his paper, labelled as ‘No Cheating’.

**Table 4:** Hyper parameters of the models [37].

Parameter	Value/Name
Batch size	1
Max_Proposals	300
iou_threshold	0.7
Momentum optimizer value	0.9
Localization_loss_weight	1.0
Kernal_size	2
Score_Converter	Softmax
Num-steps	60,000
Num_examples	899
Max eval	10
Loss function Learning rate	MSE 0.0002

**Table 5:** Confusion Matrix dipicting cheating and no cheating prediction.

	FP	TP
Cheating	590	8
No Cheating	4	398
	TN	FN

Where TP, FN, FP, and TN represent the number of true positives, false negatives, false positives, and true negatives, respectively.

**Table 6:** Comparison Table for Accuracy.

Activity	Correctly Identified	Total	Accuracy
Cheating	590	600	98.3
No Cheating	398	400	99.5

## Data Availability

The data used in this study is a real-time data.
